# A hybrid compass mechanism combining radical pairs and magnetite crystals

**DOI:** 10.1073/pnas.2524093123

**Published:** 2026-02-19

**Authors:** P. J. Hore

**Affiliations:** ^a^Department of Chemistry, University of Oxford, Oxford OX1 3QZ, United Kingdom

**Keywords:** magnetoreception, radical pair mechanism, magnetic compass sense, magnetite

## Abstract

That night-migratory songbirds have a magnetic compass sense is undisputed. The nature of the sensor, however, is far from certain. The two leading hypotheses are organic radical pairs in cryptochrome flavoproteins and crystals of inorganic magnetic minerals such as magnetite. Here, we propose a magnetoreception mechanism that combines radical pair chemistry with magnetite nanoparticles. Instead of directly detecting the tiny dependence of a radical pair reaction on the direction of the Earth’s (~50 μT) magnetic field, this hybrid sensor uses a magnetic particle to amplify the Earth’s field. Directional information is thereby encoded in the response of nearby radical pairs to the intensity of a much stronger (~5 mT) magnetic field. The result is a magnetoreceptor with potentially 10 to 100-fold greater sensitivity than afforded by radical pairs alone.

## Significance

Small night-migratory songbirds navigate thousands of kilometers assisted by an internal magnetic compass whose underlying biophysical mechanism is largely obscure. Evidence suggests that light-sensitive molecules in the birds’ eyes can respond to the Earth’s magnetic field although it is far from clear that the ensuing signal is strong enough for reliable navigation. We propose a variant of this hypothesis in which these magnetically sensitive molecules operate alongside magnetic iron oxide particles which amplify the Earth’s field. The model predicts that this partnership could increase the directional response of the sensor by up to 100-fold. By bridging biology and physics, this framework advances understanding of how migratory birds accomplish one of Nature’s most extraordinary navigational feats.

Every year billions of small songbirds fly spectacular distances between their breeding and wintering grounds, navigating with the help of an internal, light-dependent, magnetic inclination compass (1, 2). Sixty years after the discovery of this remarkable sense, the identity of the sensor and the biophysics of its action are still obscure (3, 4). Two hypotheses stand out: radical pairs and magnetic particles. The former proposes that photochemical reactions encode the direction of the Earth’s magnetic field (25 to 65 μT) via the coherent spin dynamics of cryptochrome photoreceptors (5, 6). The latter suggests that intracellular crystals of biogenic iron-containing minerals, e.g., magnetite (Fe_3_O_4_), rotate into alignment with the Earth’s field thereby opening mechanosensitive transmembrane ion channels (7, 8).

The magnetite hypothesis has solid theoretical foundations but no receptor has yet been identified, either for the map sense or the compass sense in any animal (3, 4, 7). The cryptochrome hypothesis is based on an established mechanism for the magnetic sensitivity of organic radical reactions (9). The photoreceptor protein cryptochrome 4a (CRY4a) (10–16), located in double-cone photoreceptor cells in the birds’ retinas (17), is thought to be the magnetoreceptor although much of the supporting evidence is indirect (3, 4, 18). Radical pairs formed photochemically in purified avian CRY4a are magnetically sensitive, and meet the requirements for a light-dependent sensor indifferent to the polarity of the external magnetic field (14, 19, 20). However, there are major questions about whether the in vivo response of any radical pair reaction to a magnetic field 10 to 100 times weaker than a small refrigerator magnet could be sufficient to form the basis of a viable geomagnetic sensor (21).

The low sensitivity of radical pairs to submillitesla magnetic fields can, in part, be traced back to the internal magnetic (hyperfine) interactions between electron and nuclear spins. In CRY4a, the ^1^H and ^14^N hyperfine tensors that have the largest anisotropy do not, for the most part, have mutually aligned principal axes, causing the magnetic anisotropy of the spin system as a whole to be substantially lower than that of the individual hyperfine interactions. In addition, the nuclei that have more nearly isotropic hyperfine tensors provide little directional information and tend to dilute that available from their more anisotropic neighbors. Matters are not helped by the dipolar coupling of the two electron spins (22), by spin relaxation (23, 24), by suboptimal reaction kinetics (6), or by imperfect protein alignment (25), all of which can further attenuate the magnetic response. It is unsurprising therefore that as spin dynamics simulations of radical pair magnetoreceptors are made progressively more realistic, e.g. by including more nuclear spins, so the predicted magnetic field effects become ever smaller (21, 26).

We propose here a hybrid magnetic compass mechanism that combines elements of the cryptochrome and magnetite hypotheses in a way that could afford substantially greater sensitivity than normally expected from the “standard” radical pair model, as originally described by Ritz et al. (5). The concept is distinct from earlier experimental work (27–32) and theoretical treatments (8, 33–39) of radical pairs in the neighborhood of magnetic nanostructures. Those studies focused on heterogeneous catalysis of intersystem crossing (33), magneto-fluorescence imaging (30), magnetic-field mapping (31), transduction of time-dependent magnetic fields (37, 39), and in vivo tests for a radical-pair direction sensor (38). The present proposal also differs from previous discussions of hybrid magnetic compass mechanisms which rely on either the strong magnetic-field gradient close to the surface of a nanoparticle (33, 34), the microtesla magnetic field further away from a much larger magnet (35), or the delicate balance between elastic and magnetic torques on a nanomagnet whose free rotation is hindered by cytoskeletal attachment (8, 36). Nor is it directly connected to the MagR hypothesis (40) in which CRY4 is proposed to interact with a protein whose magnetic properties appear to originate in a small number of weakly coupled iron atoms.

## Results

### Standard Radical Pair Mechanism.

Avian CRY4a is a ~64 kDa flavoprotein containing a noncovalently bound FAD (flavin adenine dinucleotide) chromophore. Blue-light irradiation excites the FAD, triggering the formation of a spin-singlet FAD^•−^ TrpH^•+^ radical pair via consecutive electron transfers along a chain of four tryptophan (TrpH) residues to the excited FAD (10–16, 19, 20). This singlet state coherently interconverts with the spin-triplet radical pair and both are transformed into a signaling state of the protein with a quantum yield that encodes the intensity and the direction of an external magnetic field via its influence on the singlet-triplet spin dynamics (5, 6). Although FAD^•−^ TrpH^•+^ accounts for the magnetic field effects observed for purified avian CRY4a (14, 20), it is conceivable that a different FAD-containing radical pair, formed during the dark reoxidation of the photoreduced protein, could be magnetically sensitive in vivo (41, 42). Given the uncertain identity and properties of this state, we focus here on FAD^•−^ TrpH^•+^, although our proposed mechanism is also applicable to these “dark” radical pairs provided their spin relaxation is not too fast (43).

**Fig. 1.**
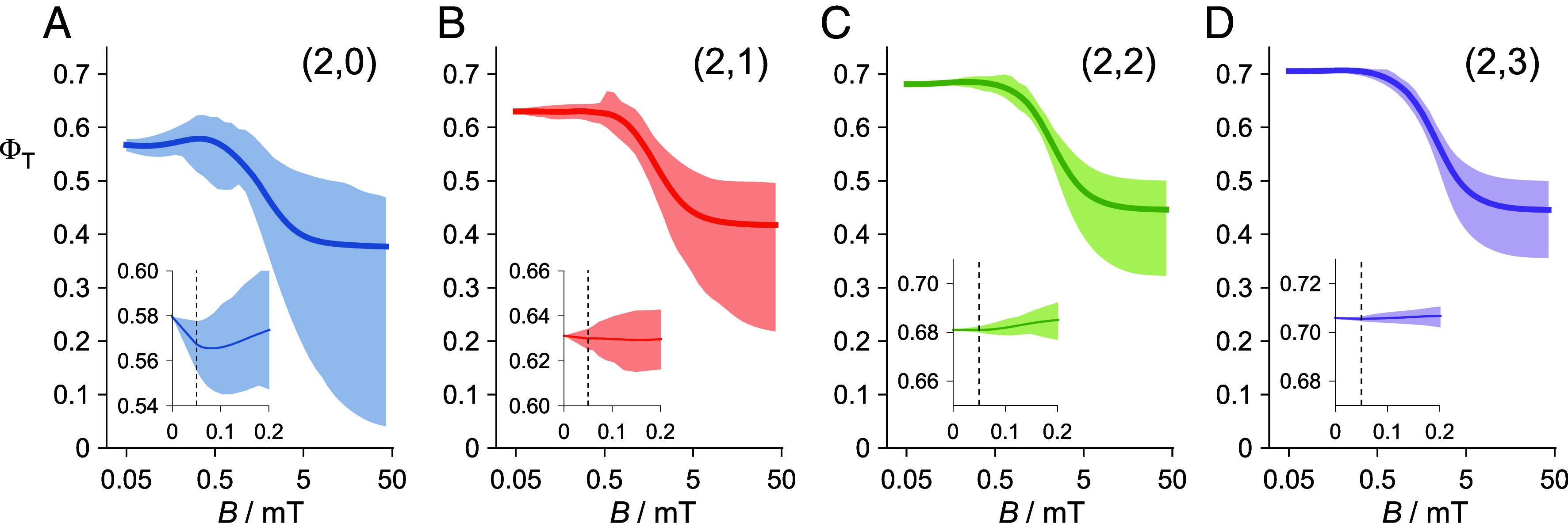
Magnetic field effects calculated for toy models of the FAD^•−^ TrpH^•+^ radical pair in avian CRY4a, with 2 nuclear spins in the flavin radical and (*A*) 0, (*B*) 1, (*C*) 2, and (*D*) 3 in the tryptophan radical. The four panels show the range (shaded areas) and average values (solid lines) of the triplet product yield for 400 directions of a magnetic field ranging from 0.05 to 50 mT. The four insets, all plotted on the same vertical scale, show the low-field regions (0 to 0.2 mT) with the geomagnetic field (0.05 mT) indicated by dashed lines.

To illustrate the sensitivity issue with the standard model and its dependence on the number of nuclear spins ($n_{\text{nuc}}$) coupled to the two electrons, Fig. 1 shows calculated magnetic field effects on toy models of FAD^•−^ TrpH^•+^. Details of these calculations are given in the *SI Appendix*, section S1. Starting with the two nuclei in FAD^•−^ that have the largest hyperfine anisotropy (Fig. 1*A*), nuclei were added one at a time to the TrpH^•+^ radical (Fig. 1 *B–D*). The four panels show the ranges (shaded areas) and averages (solid lines) of the yield of the product formed from the triplet radical pair ($\Phi_{\text{T}}$), calculated for 400 hemispherically distributed directions of a magnetic field with strength in the range *B* = 0.05-50 mT. In the following, we refer to the maximum-minus-minimum $\Phi_{\text{T}}\left(B\right)$ and the average $\Phi_{\text{T}}\left(B\right)$ as the anisotropic and isotropic signals, respectively. In all four panels, the isotropic triplet yield has the sigmoidal field dependence typical of a radical pair reaction (44). Magnetic fields comparable to or larger than the hyperfine interactions (~1 mT) suppress the formation of the T_±1_ triplet radical pair states and so reduce $\Phi_{\text{T}}$. As $n_{\text{nuc}}$ is increased, the anisotropic triplet yield shrinks across the whole range of applied fields, most markedly at low field (Fig. 1, *Insets*). For example, in the geomagnetic field (e.g., B = 50 μT), the difference between the maximum and minimum values of $\Phi_{\text{T}}$ is 0.022 for $n_{\text{nuc}}$ = 2 (Fig. 1*A*) falling to 0.0020 for $n_{\text{nuc}}$ = 5 (Fig. 1*D*) and 0.00023 for $n_{\text{nuc}}$ = 8 (*SI Appendix*, section S2). The poor detection sensitivity of the standard model is exacerbated in vivo by the low intensity of the light available to generate the radical pairs on a clear, moonless starry night [~3 photons s^−1^ μm^−2^ (6)].

The only aspect of the magnetic field effects in Fig. 1 that is not strongly attenuated as $n_{\text{nuc}}$ is increased is the isotropic effect of fields stronger than ~1 mT. The difference between $\Phi_{\text{T}}\left(50\;\text{mT}\right)$ and $\Phi_{\text{T}}\left(0\right)$ in Fig. 1 is roughly independent of the number of nuclei: 0.20 when $n_{\text{nuc}}$ = 2 (Fig. 1*A*), 0.26 when $n_{\text{nuc}}$ = 5 (Fig. 1*D*), 0.23 when $n_{\text{nuc}}$ = 8 and 0.25 when $n_{\text{nuc}}$ = 14 (*SI Appendix*, section S2). Instead of using the tiny *anisotropic* effects of the Earth’s magnetic field as the source of directional information (as in the standard radical pair model), the compass mechanism proposed here relies on the much larger isotropic magnetic field effects expected for magnetic fields between 1 and 10 mT.

### Hybrid Radical-Pair/Magnetite Mechanism.

**Fig. 2.**
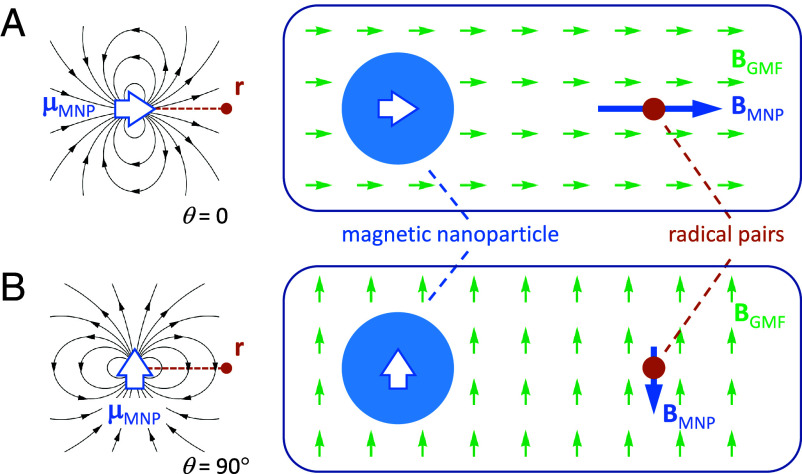
Schematic operation of the hybrid magnetoreceptor complex. In this illustration, the geomagnetic field, $B_{\text{GMF}}$ (green arrows), and the magnetic dipole moment of the nanoparticle, $\mu_{\text{MNP}}$ (open blue arrows), are parallel and make an angle θ with the vector, **r**, that defines the position of the radical pairs with respect to the center of the nanoparticle. (*A*) θ=0 and (*B*) θ=90°. $B_{\text{MNP}}$ (solid blue arrows) is the fringing field of the nanomagnet at position r. The figures on the left-hand side show the lines of magnetic flux for (*A*) θ=0 and (*B*) θ=90°.

With reference to Fig. 2, we consider an ensemble of randomly oriented CRY4a molecules at position **r** relative to the center of a single-domain magnetic nanoparticle (MNP) with a remanent magnetic dipole moment $\mu_{\text{MNP}}$. The nanoparticle is assumed to be spherical, with radius *R*. Both the radical pairs and the nanoparticle experience the geomagnetic field, $B_{\text{GMF}}$. The dipole is large enough that the magnetic energy, $\mu_{\text{MNP}}B_{\text{GMF}}$, is comparable to the thermal energy, $k_{\text{B}}T$, at physiological temperature so that $\mu_{\text{MNP}}$ tends to align with $B_{\text{GMF}}$ provided the nanoparticle is free to rotate and has no preferred orientation in the absence of an external magnetic field. Any change in the angle, θ, between $B_{\text{GMF}}$ and **r** therefore changes the average direction of $\mu_{\text{MNP}}$ relative to **r**. The radical pairs therefore experience a magnetic field $B_{\text{GMF}}+B_{\text{MNP}}\left(r\right)$, where $B_{\text{MNP}}\left(r\right)$ is the fringing field of the nanomagnet:

[1] $$\begin{array}{c} B_{\text{MNP}}\left(r\right)\;=\;\left(\frac{\mu_{0}}{4\pi }\right)\left(-\frac{\mu_{\text{MNP}}}{r^{3}}+\frac{3\left(\mu_{\text{MNP}}.r\right)r}{r^{5}}\right), \end{array}$$

and $\mu_{0}$ is the vacuum permeability, $4\pi \times 10^{-7}\;\text{H}\;{\text{m}}^{-1}$. When $\mu_{\text{MNP}}$ is parallel to $B_{\text{GMF}}$ and both are parallel to **r** (θ=0, Fig. 2*A*), $B_{\text{MNP}}\left(r\right)$ is parallel to $B_{\text{GMF}}$. When $\mu_{\text{MNP}}$ and $B_{\text{GMF}}$ are perpendicular to **r** (θ=90°, Fig. 2*B*), $B_{\text{MNP}}\left(r\right)$ is antiparallel to $B_{\text{GMF}}$ and half the size it was when θ=0. The magnitude of the net magnetic field at position **r**, $\left|B_{\text{GMF}}+B_{\text{MNP}}\left(r\right)\right|$, therefore depends on the direction of $B_{\text{GMF}}$ relative to the nanoparticle–CRY4a axis, **r**. The direction of $B_{\text{GMF}}+B_{\text{MNP}}\left(r\right)$ also depends on *θ* but that has no relevance here because the radical pairs are randomly oriented so that any anisotropic magnetic field effect will not survive ensemble averaging. If **r** is not too large, $B_{\text{MNP}}\left(r\right)$ can be substantially stronger than $B_{\text{GMF}}$. Thus, the magnetic nanoparticle can be said to amplify $B_{\text{GMF}}$ in a manner that encodes its direction. We refer to the combination of nanomagnetic amplifier and radical-pair sensor in Fig. 2 as the “magnetoreceptor complex.”

**Fig. 3.**
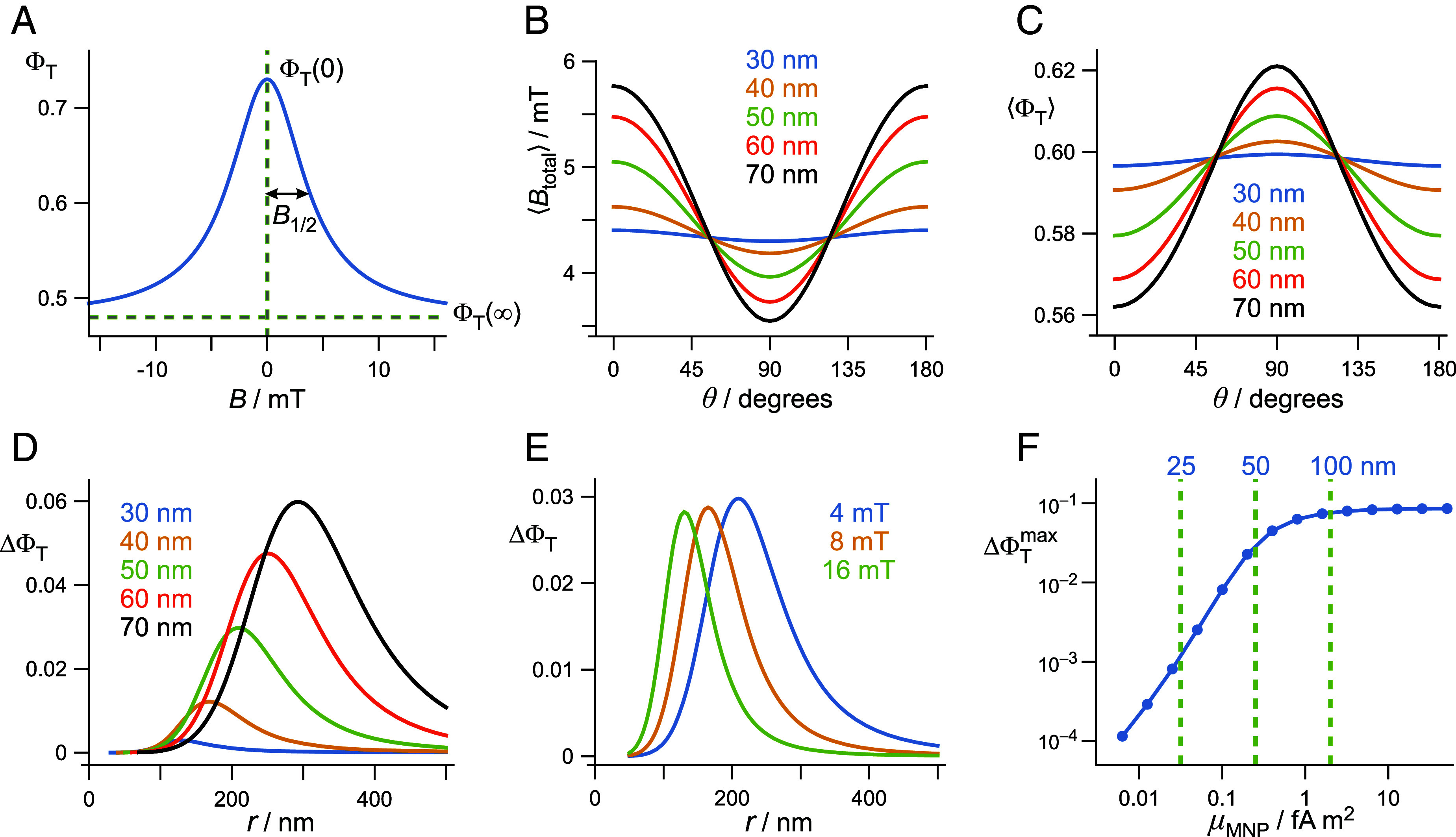
(*A*) Magnetic field dependence of the triplet product yield, Eq. **2**, with $\Phi_{\text{T}}\left(0\right)$ = 0.73, $\Phi_{\text{T}}\left(\infty \right)$ = 0.48, $B_{1/2}$ = 4 mT. These values were used for all six panels with the exception of $B_{1/2}$ in panel (*E*). (*B*) Dependence of the averaged magnetic field, Eq. **3**, experienced by radical pairs positioned at r=4R (with *R* = 30 to 70 nm), on the angle between the geomagnetic field and the vector **r**. (*C*) Averaged triplet yields under the conditions of panel (*B*). (*D*) Dependence of $\Delta \Phi_{\text{T}}$, Eq. **4**, on the distance of the radical pairs from a magnetic nanoparticle of radius *R* = 30 to 70 nm. (*E*) Dependence of $\Delta \Phi_{\text{T}}$, Eq. **4**, on the distance of the radical pairs from a magnetic nanoparticle of radius *R* = 50 nm, with $B_{1/2}$ = 4, 8, 16 mT. (*F*) Dependence of the maximum $\Delta \Phi_{\text{T}}$ on the magnetic dipole moment of the nanoparticle. The dashed lines indicate the values of $\mu_{\text{MNP}}$ for spherical particles of radius *R* = 25, 50, 100 nm. Details of all these calculations are given in the *SI Appendix*, section S3.

To explore the sensitivity of this hybrid magnetoreceptor complex, we model the field dependence of $\Phi_{\text{T}}$ for the ensemble of CRY4a molecules as a Lorentzian with half-width at half-maximum height, $B_{1/2}$ = 4 mT (Fig. 3*A*):

[2] $$\begin{array}{c} \Phi_{\text{T}}\left(B\right)=\Phi_{\text{T}}\left(\infty \right)+\frac{\Phi_{\text{T}}\left(0\right)-\Phi_{\text{T}}\left(\infty \right)}{1+{\left(B/B_{1/2}\right)}^{2}}. \end{array}$$

Both the Lorentzian form and this value of $B_{1/2}$ are typical for FAD^•−^ TrpH^•+^ radical pairs in purified avian CRY4a (14, 19, 20). The magnitude of the magnetic field effect is determined by the values of $\Phi_{\text{T}}\left(0\right)$ and $\Phi_{\text{T}}\left(\infty \right)$ (Fig. 3*A*). For reasons that will shortly become clear, we use $\Phi_{\text{T}}\left(0\right)$ = 0.73 and $\Phi_{\text{T}}\left(\infty \right)$ = 0.48.

We consider the nanoparticle to be composed of magnetite with a saturation magnetization, $M_{\text{S}}$ = 4.8 × 10^5^ A m^−1^ (37), and magnetic dipole moment, $\mu_{\text{MNP}}=\left(4\pi R^{3}/3\right)M_{\text{S}}$. The condition for $\mu_{\text{MNP}}$ to be significantly aligned with the geomagnetic field is $\eta =\mu_{\text{MNP}}B_{\text{GMF}}/k_{\text{B}}T$ > 1. When $B_{\text{GMF}}$ = 50 μT and *T* = 40 °C (physiological temperature), η > 1 when $\mu_{\text{MNP}}$ > 0.086 fA m^2^ and hence when the particle radius, *R*, is greater than 35 nm.

For the purposes of the proposed detection scheme, the magnetic fields experienced by the radical pairs should correspond to the part of Fig. 3*A* where $\Phi_{\text{T}}\left(B\right)$ has the strongest dependence on *B*, i.e. 1 < *B* < 10 mT. If $\mu_{\text{MNP}}$ were aligned perfectly with $B_{\text{GMF}}$ (i.e., when η≫1), this would occur at a position r≈4R where 3.14 ≤ $B_{\text{MNP}}\left(r\right)$ ≤ 6.28 mT, depending on the direction of $B_{\text{GMF}}$ and therefore $\mu_{\text{MNP}}$. Under these conditions, the magnetic field at the position of the radical pairs would be $60B_{\text{GMF}}\;\mathrm{to}\;120B_{\text{GMF}}$, independent of *R*. For smaller values of η, $\mu_{\text{MNP}}$ is less well aligned with $B_{\text{GMF}}$ and the net magnetic field at r is an average, $\langle \cdots \rangle$, over the distribution of $\mu_{\text{MNP}}$ directions:

[3] $$\begin{array}{c} \langle B_{\text{total}}(r,\theta )\rangle \;\;=\;\;\langle \left|B_{\text{GMF}}+B_{\text{MNP}}(r)\right|\;\rangle , \end{array}$$

(*SI Appendix*, section S3). Fig. 3*B* shows the dependence of $\langle B_{\text{total}}\left(4R,\theta \right)\rangle$ on *θ*, for five nanoparticle radii. In all five cases, $\langle B_{\text{total}}\left(r,\theta \right)\rangle$ is substantially larger than $B_{\text{GMF}}$, with the larger nanoparticles producing a stronger orientation dependence because of the greater alignment of $\mu_{\text{MNP}}$ with $B_{\text{GMF}}$.

Using Eq. **2**, Fig. 3*C* shows the dependence of the averaged triplet yield, $\langle \Phi_{\text{T}}\left(B_{\text{total}}\left(4R,\theta \right)\right)\rangle$, on θ under the conditions of Fig. 3*B*. The triplet yield depends on the direction of $B_{\text{GMF}}$ for the same reason that $\langle B_{\text{total}}\left(4R,\theta \right)\rangle$ does. Fig. 3 *B* and *C* resemble one another because $\Phi_{\text{T}}\left(B\right)$ is approximately linear in *B* in the relevant field range (3.14 to 6.28 mT, Fig. 3*A*).

The dependence of $\langle \Phi_{\text{T}}\rangle$ on the direction of $B_{\text{GMF}}$ (i.e., on θ) is assumed to provide the signal from which a bird could derive a magnetic compass bearing. This quantity, defined here as

[4] $$\begin{array}{c} \Delta \Phi_{\text{T}}=\langle \Phi_{\text{T}}\left(B_{\text{total}}(r,90{}^{\circ})\right)\rangle -\langle \Phi_{\text{T}}\left(B_{\text{total}}(r,0)\right)\rangle , \end{array}$$

is plotted against *r* in Fig. 3*D* using the same nanoparticle radii as in Fig. 3 *B* and *C*. As *R* and therefore $\mu_{\text{MNP}}$ are increased, $\mu_{\text{MNP}}$ is more closely aligned with $B_{\text{GMF}}$, and the signal, $\Delta \Phi_{\text{T}}$, increases. The maxima in Fig. 3*D* occur at roughly four times the nanoparticle radii as anticipated above. As shown in Fig. 3*E*, $\Delta \Phi_{\text{T}}$ is not strongly dependent on $B_{1/2}$: As the width of the field-dependence (Fig. 3*A*) is increased, the radical pairs need to be closer to the nanoparticle to achieve the maximum $\Delta \Phi_{\text{T}}$. Fig. 3 *D*–*F* were drawn for specific values of $\Phi_{\text{T}}\left(0\right)$ and $\Phi_{\text{T}}\left(\infty \right)$ but can easily be generalized using the proportionality between $\Delta \Phi_{\text{T}}$ and $\Phi_{\text{T}}\left(0\right)-\Phi_{\text{T}}\left(\infty \right)$ (Eqs. **2** and **4**).

Finally, Fig. 3*F* shows how $\Delta \Phi_{\text{T}}^{\text{max}}$, the maximum value of $\Delta \Phi_{\text{T}}$ (e.g. in Fig. 3*D*), varies with the magnetic dipole moment of the nanoparticle. As $\mu_{\text{MNP}}$ is increased, $\mu_{\text{MNP}}$ becomes more closely aligned with $B_{\text{GMF}}$ and so $\Delta \Phi_{\text{T}}^{\text{max}}$ increases. Once $\mu_{\text{MNP}}$ is greater than ~1 fA m^2^, the alignment is almost perfect, and the signal reaches a plateau ($\Delta \Phi_{\text{T}}^{\text{max}}$ = 0.17, *SI Appendix*, section S3).

## Discussion

The potential sensitivity enhancement of this hybrid compass relative to the standard model can be estimated by means of spin dynamics simulations of FAD^•−^ TrpH^•+^ (*SI Appendix*, section S2). Including the 14 nuclear spins with the largest hyperfine interactions (7 in FAD^•−^ and 7 in TrpH^•+^), the anisotropy of the reaction yield in a 50-μT magnetic field is 8.5 × 10^−5^ in the standard model. To obtain the corresponding figure for the hybrid model, we first determined the isotropic triplet yield in zero field and in a magnetic field much stronger than the hyperfine interactions: $\Phi_{\text{T}}\left(0\right)$ = 0.73, $\Phi_{\text{T}}\left(\infty \right)$ = 0.48, and hence $\Phi_{\text{T}}\left(0\right)-\Phi_{\text{T}}\left(\infty \right)$ = 0.25 (*SI Appendix*, section S2). These are precisely the values that were used for Fig. 3 *A*–*F*. Therefore, we can simply read off from Fig. 3*F* the maximum signal for a *R* = 50 nm spherical nanoparticle ($\mu_{\text{MNP}}$ = 0.25 fA m^2^) as $\Delta \Phi_{\text{T}}^{\max}$ = 0.030. The estimated sensitivity gain of the hybrid model over the standard model is therefore 0.03/(8.5 × 10^−5^) = 350. The corresponding enhancements for *R* = 40, 30, and 20 nm ($\mu_{\text{MNP}}$ = 0.13, 0.054, and 0.016 fA m^2^) are 140, 34, and 4.9. When $\mu_{\text{MNP}}$ > 1 fA m^2^, the alignment of $\mu_{\text{MNP}}$ with $B_{\text{GMF}}$ is almost perfect (Fig. 3*F*) and the enhancement plateaus at just over 1,000. These potential improvements in sensitivity could be even larger given the differential effects of spin relaxation at high and low field, which were excluded from these simulations (*SI Appendix*, section S4).

It is currently impossible to derive an alternative estimate of the sensitivity gain using exclusively experimental data because no anisotropic magnetic field effects at 50 μT have yet been measured for any cryptochrome. However, an approximate value can be obtained from the 8% isotropic magnetic field effect reported for European robin CRY4a (14). Taking $\Phi_{\text{T}}\left(0\right)$ = 0.73, as above, an 8% effect corresponds to $\Phi_{\text{T}}\left(0\right)-\Phi_{\text{T}}\left(\infty \right)$ ≈ 0.06, i.e., 4.2 times smaller than the value (0.25) obtained from the simulations mentioned in the last paragraph. Since $\Delta \Phi_{\text{T}}$ is proportional to $\Phi_{\text{T}}\left(0\right)-\Phi_{\text{T}}\left(\infty \right)$, we can estimate a sensitivity enhancement of 350/4.2 ≈ 80 for a *R* = 50 nm nanoparticle. This result may also be an underestimate because the attenuating effects of spin relaxation are included in the experimentally measured (8%) magnetic field effect but not in the standard-model simulations.

To put these estimates in context, an *N*-fold increase in the signal strength translates to an $N^{2}$-fold reduction in the number of radical pairs required to achieve a compass bearing with a given precision (e.g., to within 5°, *SI Appendix*, section S5) (21, 45). A hybrid compass could therefore function satisfactorily with fewer radical pairs, and therefore lower light intensities, than a standard-model compass. A rough estimate of the number of photons that enter the double-cone photoreceptor cells in a songbird’s eye on a clear, moonless, star-lit night is ~2 × 10^8^ min^−1^ (21). Assuming the ~350-fold enhancement estimated above, this number is roughly 1,000 times more than would be required for a precision of 5° using a hybrid compass and about 100 times too few if using a standard-model compass. *SI Appendix*, sections S6 and S7 summarize the origins of the sensitivity differences between the two models.

Previous discussions of the effects of nanomagnets on radical pair reactions have concentrated on singlet-triplet interconversion driven by the gradient of the fringing field (33, 34). Such effects are not expected to be important here (*SI Appendix*, section S8).

Despite their differences, the two models have a number of important aspects in common. The hybrid model still requires blue light to excite the cryptochrome and, like the standard model, is an inclination compass (1, 26). Although the direction of the total field experienced by the radical pairs changes when $B_{\text{GMF}}$ is inverted, its magnitude does not and, as explained above, it is the strength of the total field that encodes the direction of $B_{\text{GMF}}$. The two models both assume that the compass bearing is transduced via a signaling state of the protein interacting with partner proteins to initiate a signal transduction cascade with biochemical amplification. In principle, both models are compatible with the proposed “dark” radical pair, ${\text{FADH}}^{∙}\;{\text{O}}_{2}^{∙-}$ (41, 42), but only if Nature has found a way to circumvent the extremely fast spin relaxation of the superoxide radical (43).

An essential feature of the standard model is that the cryptochromes in each cell should be mutually aligned (25, 45). Throughout the avian retina, CRY4a is found in the outer segments of the long-wavelength and double-cone photoreceptor cells (17, 46), the latter being a favorable location for a magnetic direction sensor. Approximately cylindrical and pointing toward the pupil, the outer segments of the double cones are packed with oriented membranes carrying visual pigment proteins (iodopsins) to which the cryptochromes could be tethered, and thereby aligned and immobilized (17). Double cones at different locations in the approximately hemispherical retina have different orientations and should therefore have different but correlated responses to the geomagnetic field. Processing of signals from cells distributed across the retina should improve signal-to-noise and provide the information the bird would need to determine a magnetic compass bearing (21, 45). The same principles apply in the hybrid model except that it is the magnetoreceptor complexes that must be aligned within the cells so that cells at different locations once again transduce correlated directional information. Although the cryptochromes must not be uniformly distributed around the nanomagnet, they do not have to be located at a precise distance from it. For example, if the CRY4a molecules occupy a spherical region with radius 50 nm, centered 210 nm away from a *R* = 50 nm nanoparticle, $\Delta \Phi_{\text{T}}$ would be 89% of its value when all the sensors are localized at 210 nm. This percentage drops to 66% if the CRY4a “pool” has a radius of 100 nm (*SI Appendix*, section S9). Thus, it is not crucial for all the sensors to have the same distance from the amplifier within the magnetoreceptor complex. Although we have presented the hybrid model in terms of randomly oriented CRY4a molecules, it would work just as well if the proteins were partially aligned via their interactions with membrane proteins. Each cell could contain several magnetoreceptor complexes for improved signal-to-noise.

A potential difficulty with both models is that the fraction of the cryptochrome molecules present in the signaling state in a cell will depend much more strongly on the intensity and possibly polarization of the incoming light than on the direction of the geomagnetic field experienced by the cell (47). A possible solution to this problem is to compare the signals from nearest neighbor cells which would experience the same photon flux but would have different magnetic responses (47). Variations in both light intensity and polarization could be canceled if adjacent cells were differentially rotated around the retina normal. Subsequently, it was found that the double cones form an oriented mosaic that could enable just such a cancelation to occur (46). This mechanism, derived with the standard model in mind, should apply equally well to the hybrid model provided the magnetite-CRY4a vector r has a component perpendicular to the rotation axis of the cells.

As argued above, a hybrid magnetite-cryptochrome magnetic compass offers a major sensitivity advantage. On the basis of Fig. 3*F*, the hybrid design would deliver directional signals 10 to 100 times larger than the standard model if the nanoparticle had a magnetic dipole moment in the range 0.03 to 0.10 fA m^2^, corresponding to a radius of 20 to 40 nm for a spherical nanomagnet or to a length of 60 to 100 nm for a cuboidal particle with length/width ratio equal to 2. These dimensions fall within the range in which magnetite crystals are expected to be single-domain rather than superparamagnetic or multidomain (48). They are also comparable to the single-domain magnetite crystals found in biological tissue [typically ~50 nm (49)] and to the intracellular magnetite crystals contained in the magnetosomes of magnetotactic bacteria [typically 30 to 140 nm (50)]. An enhancement greater than ~500 would require a magnetic dipole moment (>0.4 fA m^2^) outside the normal range for single-domain magnetite crystals. Such large magnetic moments might arise from compact clusters of several smaller crystals (51).

Although there seem to be no confirmed reports of intracellular magnetite crystals in the avian retina, small magnetite particles may have escaped detection. An extensive study, using rotating magnetic fields to detect spinning cells, revealed no biogenic magnetite in pigeon tissue (52). However, this assay would not have been sensitive to particles with the relatively small magnetic moments (<1 fA m^2^, Fig. 3*F*) needed for the hybrid model. Moreover, cells containing the loosely anchored magnetite crystals required here, would not have been induced to spin whatever their magnetic moments.

In addition to a sufficient magnetic moment, the other main requirement for the nanoparticle is that it is able to rotate relatively freely into alignment with the Earth’s magnetic field. Its rotational motion should therefore not be constrained by attachment to cellular structures [as required in an earlier hybrid compass proposal (36)]. A simple treatment of the magnetic and viscous torques experienced by a spherical magnetic particle suspended in an isotropic fluid (53) suggests that the characteristic time taken to align with the Earth’s field is independent of the particle radius, proportional to viscosity, and equal to ~200 μs for water at 40 °C (*SI Appendix*, section S10). Even if the surrounding medium had ten times the viscosity of water, alignment would be complete within ~10 ms. It seems unlikely that a bird would need to determine a compass bearing more rapidly than that. Indeed, it could be advantageous, for signal-to-noise reasons, to integrate or otherwise accumulate directional information for much longer periods (e.g. ~1 min) (21, 45).

Perhaps the most serious piece of evidence against this hybrid compass mechanism in migratory songbirds is that it is incompatible with recent behavioral tests using radiofrequency magnetic fields superimposed on the geomagnetic field. The finding that Eurasian blackcaps were unable to use their magnetic compass when subjected to (~ 1 pT/$\sqrt{\text{Hz}}$) broadband magnetic noise in the frequency bands 1 to 10 MHz and 80 ± 5 MHz (54) but were unaffected by 140 ± 5 MHz and 240 ± 5 MHz fields of similar intensity (55) is qualitatively consistent with the standard radical pair hypothesis in which a FAD^•−^-containing radical pair acts as the sensor. This conclusion was based on the calculated maximum frequency of singlet-triplet interconversion in the FAD^•−^ TrpH^•+^ radical pair (116 MHz) which is the predicted threshold between disorientation at lower frequencies and no effect at higher frequencies (55, 56). A hybrid compass, in which the radicals experience a static magnetic field approximately 100 times stronger than the geomagnetic field, would have faster singlet-triplet spin dynamics and therefore a higher threshold for radiofrequency disorientation that would be inconsistent with the behavioral tests (*SI Appendix*, section S11). However, it would be premature to dismiss the possibility of a hybrid compass mechanism given that the standard model is quantitatively unable to account for the disorientation caused by radiofrequency fields predicted to be at least 1,000 times too weak to produce significant changes in the yield of a cryptochrome signaling state (56).

Finally, how could a hybrid direction sensor be distinguished from one based on the standard model? Short, intense magnetic-field pulses have been extensively used in behavioral tests as a diagnostic for magnetite-based magnetic sensors (57–64). However, the hybrid sensor proposed here should not be affected by pulsed magnetic fields because it relies on a single untethered magnetite crystal rather than a chain of interacting particles attached to a cytoskeletal structure. If the experiment involves a static, biasing magnetic field (61–63), the nanoparticle would rotate to align its magnetic moment with the field. A strong magnetic pulse parallel to the bias field should then have no effect while an antiparallel field should invert the magnetic moment of the particle. As soon as the two magnetic fields are switched off, however, the particle would quickly rotate so that its magnetic moment aligns once more with the Earth’s magnetic field. The net change to the particle would be zero. The same would be true if the test were performed without a bias field.

**Fig. 4.**
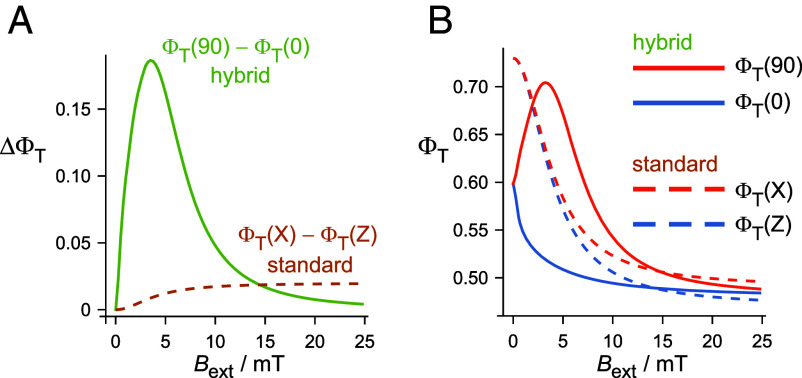
Dependence of (*A*) $\Delta \Phi_{\text{T}}$ and (*B*) $\Phi_{\text{T}}$ for the standard and hybrid models on the strength of an external magnetic field, $B_{\text{ext}}$. For the hybrid model, $\Delta \Phi_{\text{T}}$ is the difference between $\Phi_{\text{T}}\left(\theta =90{}^{\circ}\right)$ and $\Phi_{\text{T}}\left(\theta =0\right)$ (Eq. **4** and Fig. 2). For the standard model, $\Delta \Phi_{\text{T}}=\Phi_{\text{T}}\left(\text{X}\right)-\Phi_{\text{T}}\left(\text{Z}\right)$, where X and Z indicate the direction of the external field relative to the axis system of the flavin radical (*SI Appendix*, section S2). All calculations use Eq. **2** with $\Phi_{\text{T}}\left(0\right)$ = 0.73 and $B_{1/2}$ = 4 mT. $\Phi_{\text{T}}\left(\infty \right)$ = 0.48 (hybrid model), 0.49 (standard model, X), or 0.47 (standard model, Z). R = 25 nm and r=4R. Hybrid sensors with different nanoparticle radii (*R*) have the $\Delta \Phi_{\text{T}}$-maximum at similar values of $B_{\text{ext}}$ (3 to 4 mT).

More promising approaches are suggested by the predicted responses of the two sensors to external magnetic fields, $B_{\text{ext}}$, stronger than the Earth’s. Fig. 4*A* compares the directional signals ($\Delta \Phi_{\text{T}}$) expected for the two models as a function of $B_{\text{ext}}$. The standard model shows the sigmoidal dependence characteristic of radical pair reactions, leveling out when $B_{\text{ext}}$ exceeds $B_{1/2}$. By contrast, a hybrid sensor, based on the same radical pairs, is predicted to have a peaked response with a pronounced maximum at 3 to 4 mT. The origin of this striking difference can be seen from the signals ($\Phi_{\text{T}}$) predicted for orthogonal external magnetic fields, shown Fig. 4*B*. Note that each of the $\Delta \Phi_{\text{T}}$ traces in Fig. 4*A* is the difference between the corresponding parallel and perpendicular traces in Fig. 4*B*. In the case of the standard sensor, the signals for applied fields parallel and perpendicular to the flavin *Z*-axis are rather similar, reflecting the relatively small anisotropy afforded by the hyperfine interactions (Fig. 1 and *SI Appendix*, section S2). For the hybrid model, when $B_{\text{ext}}$ is parallel to the vector **r** (*θ* = 0), the field generated by the nanoparticle, $B_{\text{MNP}}$, is roughly parallel to $B_{\text{ext}}$ so that the proteins experience a net field stronger than $B_{\text{ext}}$ (geometry as in Fig. 2*A*). Conversely, when $B_{\text{ext}}$ is perpendicular to **r** (θ=90°), $B_{\text{MNP}}$ is antiparallel to $B_{\text{ext}}$ and the net field at the position of the cryptochromes is smaller than $B_{\text{ext}}$ (geometry as in Fig. 2*B*). The form of Fig. 4 can then be understood by noting that smaller net fields correspond to larger $\Phi_{\text{T}}$ (Fig. 3*A*).

The pronounced difference in the signals predicted for the two types of sensor in the range $1\;\text{mT}\leq B_{\text{ext}}\leq 10\;\text{mT}$ (Fig. 4*A*) should allow the mechanisms to be distinguished. One possibility could be to use in vivo or ex vivo electrophysiology (65–68). However, previous attempts to detect a dependence of neuronal activity on the direction of the geomagnetic field were either irreproducible or have yet to be independently replicated. A more promising approach would be two-photon calcium imaging (69–71), again in vivo or ex vivo. Such methods would not suffer from the magnetically induced electrical artefacts which have bedeviled electrophysiological recordings. Finally, it should be possible to adapt the fluorescence-based methods that have been used to detect magnetic field effects on purified cryptochromes (14, 20, 72, 73) for measurements on the photoreceptor cells that appear to contain the magnetoreceptors. The difficulties of using behavioral tests to distinguish the two models are discussed briefly in the *SI Appendix*, section S12.

## Materials and Methods

The toy-model data in Fig. 1 were calculated as described in ref. 74. Calculations for two larger models of the flavin–tryptophan radical pair were performed using the *MolSpin* package (24, 75), either by exactly solving the Liouville-von Neumann equation ($n_{\text{nuc}}=8$) (75) or by using the stochastic Schrödinger equation method ($n_{\text{nuc}}\text{=}\text{14}$) (24, 75, 76). Hyperfine interactions were taken from the Supplementary Information of Ref. 44. See *SI Appendix*, sections S1 and S2 for further details. Eqs. **1**–**4** were used to produce the data in Figs. 3 and 4, as described in the *Results* and in *SI Appendix*, section S3.

## Supplementary Material

Appendix 01 (PDF)

## Data Availability

All study data are included in the article and/or *SI Appendix*.

## References

[1] R. Wiltschko, W. Wiltschko, Magnetic Orientation in Animals (Springer Verlag, Berlin, 1995).

[2] H. Mouritsen, Long-distance navigation and magnetoreception in migratory animals. Nature **558**, 50–59 (2018). 29875486 10.1038/s41586-018-0176-1

[3] S. Nimpf, D. A. Keays, Myths in magnetosensation. iScience **25**, 104454 (2022). 35677648 10.1016/j.isci.2022.104454 PMC9167971

[4] G. C. Nordmann, T. Hochstoeger, D. A. Keays, Magnetoreception - A sense without a receptor. PLoS Biol. **15**, e2003234 (2017). 29059181 10.1371/journal.pbio.2003234 PMC5695626

[5] T. Ritz, S. Adem, K. Schulten, A model for photoreceptor-based magnetoreception in birds. Biophys. J. **78**, 707–718 (2000). 10653784 10.1016/S0006-3495(00)76629-X PMC1300674

[6] P. J. Hore, H. Mouritsen, The radical pair mechanism of magnetoreception. Annu. Rev. Biophys. **45**, 299–344 (2016). 27216936 10.1146/annurev-biophys-032116-094545

[7] J. Shaw , Magnetic particle-mediated magnetoreception. J. R. Soc. Interface **12**, 20150499 (2015). 26333810 10.1098/rsif.2015.0499 PMC4614459

[8] M. Winklhofer, J. L. Kirschvink, A quantitative assessment of torque-transducer models for magnetoreception. J. Roy. Soc. Interface **7**, S273–S289 (2010). 20086054 10.1098/rsif.2009.0435.focus PMC2843997

[9] P. J. Hore, Magneto-oncology: A radical pair primer. Front. Oncol. **15**, 1539718 (2025). 40123899 10.3389/fonc.2025.1539718 PMC11925880

[10] N. Ozturk , Comparative photochemistry of animal type 1 and type 4 cryptochromes. Biochemistry **48**, 8585–8593 (2009). 19663499 10.1021/bi901043s PMC2739604

[11] H. Mitsui , Overexpression in yeast, photocycle, and in vitro structural change of an avian putative magnetoreceptor cryptochrome 4. Biochemistry **54**, 1908–1917 (2015). 25689419 10.1021/bi501441u

[12] B. D. Zoltowski , Chemical and structural analysis of a photoactive vertebrate cryptochrome from pigeon. Proc. Natl. Acad. Sci. U.S.A. **116**, 19449–19457 (2019). 31484780 10.1073/pnas.1907875116 PMC6765304

[13] T. Hochstoeger , The biophysical, molecular, and anatomical landscape of pigeon CRY4: A candidate light-based quantal magnetosensor. Sci. Adv. **6**, eabb9110 (2020). 32851187 10.1126/sciadv.abb9110 PMC7423367

[14] J. Xu , Magnetic sensitivity of cryptochrome 4 from a migratory songbird. Nature **594**, 535–540 (2021). 34163056 10.1038/s41586-021-03618-9

[15] H. Otsuka , Rapid oxidation following photoreduction in the avian cryptochrome 4 photocycle. Biochemistry **59**, 3615–3625 (2020). 32915550 10.1021/acs.biochem.0c00495

[16] D. Timmer , Tracking the electron transfer cascade in European robin cryptochrome 4 mutants. J. Am. Chem. Soc. **145**, 11566–11578 (2023). 37195086 10.1021/jacs.3c00442 PMC10236492

[17] A. Günther , Double-cone localization and seasonal expression pattern suggest a role in magnetoreception for European robin cryptochrome 4. Curr. Biol. **28**, 211–223 (2018). 29307554 10.1016/j.cub.2017.12.003

[18] Y. Vortman, R. Fitak, E. Natan, Magnetoreception and the ruling hypothesis. J. Exp. Biol. **228**, jeb250252 (2025). 40207401 10.1242/jeb.250252

[19] M. Golesworthy , Singlet-triplet dephasing in radical pairs in avian cryptochromes leads to time-dependent magnetic field effects. J. Chem. Phys. **159**, 105102 (2023). 37694754 10.1063/5.0166675

[20] J. Gravell , Spectroscopic characterisation of radical pair photochemistry in nonmigratory avian cryptochromes: Magnetic field effects in *GgCry4a*. J. Am. Chem. Soc. **147**, 24286–24298 (2025). 40587190 10.1021/jacs.4c14037 PMC12272691

[21] H. G. Hiscock , Navigating at night: Fundamental limits on the sensitivity of radical pair magnetoreception under dim light. Q. Rev. Biophys. **52**, e9 (2019). 31637984 10.1017/S0033583519000076

[22] N. S. Babcock, D. R. Kattnig, Electron-electron dipolar interaction poses a challenge to the radical pair mechanism of magnetoreception. J. Phys. Chem. Lett. **11**, 2414–2421 (2020). 32141754 10.1021/acs.jpclett.0c00370 PMC7145362

[23] D. R. Kattnig, I. A. Solov’yov, P. J. Hore, Electron spin relaxation in cryptochrome-based magnetoreception. Phys. Chem. Chem. Phys. **18**, 12443–12456 (2016). 27020113 10.1039/c5cp06731f

[24] L. Gerhards, C. Nielsen, D. R. Kattnig, P. J. Hore, I. A. Solov’yov, Modeling spin relaxation in complex radical systems using MolSpin. J. Comput. Chem. **44**, 1704–1714 (2023). 37186467 10.1002/jcc.27120

[25] J. C. S. Lau, N. Wagner-Rundell, C. T. Rodgers, N. J. B. Green, P. J. Hore, Effects of disorder and motion in a radical pair magnetoreceptor. J. Roy. Soc. Interface **7**, S257–S264 (2010). 20007172 10.1098/rsif.2009.0399.focus PMC2844003

[26] P. L. Benjamin, L. Gerhards, I. A. Solov’yov, P. J. Hore, Magnetosensitivity of model flavin-tryptophan radical pairs in a dynamic protein environment. J. Phys. Chem. B **129**, 5937–5947 (2025). 40464336 10.1021/acs.jpcb.5c01187 PMC12183739

[27] P. Herve, F. Nome, J. H. Fendler, Magnetic effects on chemical-reactions in the absence of magnets - Effects of surfactant vesicle entrapped magnetite particles on benzophenone photochemistry. J. Am. Chem. Soc. **106**, 8291–8292 (1984).

[28] J. C. Scaiano, S. Monahan, J. Renaud, Dramatic effect of magnetite particles on the dynamics of photogenerated free radicals. Photochem. Photobiol. **65**, 759–762 (1997).

[29] C. F. Chignell, R. H. Sik, Effect of magnetite particles on photoinduced and nonphotoinduced free radical processes in human erythrocytes. Photochem. Photobiol. **68**, 598–601 (1998). 9796445

[30] N. Yang, A. E. Cohen, Optical imaging through scattering media via magnetically modulated fluorescence. Opt. Express **18**, 25461–25467 (2010). 21164893 10.1364/OE.18.025461

[31] H. Lee, N. Yang, A. E. Cohen, Mapping nanomagnetic fields using a radical pair reaction. Nano Lett. **11**, 5367–5372 (2011). 22044347 10.1021/nl202950h

[32] N. G. Chalkias, P. Kahawong, E. P. Giannelis, Activity increase of horseradish peroxidase in the presence of magnetic particles. J. Am. Chem. Soc. **130**, 2910–2911 (2008). 18275197 10.1021/ja7102263

[33] A. E. Cohen, Nanomagnetic control of intersystem crossing. J. Phys. Chem. A **113**, 11084–11092 (2009). 19725575 10.1021/jp907113p

[34] J. Cai, Quantum probe and design for a chemical compass with magnetic nanostructures. Phys. Rev. Lett. **106**, 100501 (2011). 21469779 10.1103/PhysRevLett.106.100501

[35] Y. Lü, T. Song, Avian magnetoreception model realized by coupling a magnetite-based mechanism with a radical-pair-based mechanism. Chin. Phys. B **22**, 048701 (2013).

[36] V. N. Binhi, Stochastic dynamics of magnetosomes and a mechanism of biological orientation in the geomagnetic field. Bioelectromagnetics **27**, 58–63 (2006). 16283662 10.1002/bem.20178

[37] V. Binhi, Do naturally occurring magnetic nanoparticles in the human body mediate increased risk of childhood leukaemia with EMF exposure? Int. J. Radiat. Biol. **84**, 569–579 (2008). 18661373 10.1080/09553000802195323

[38] S. B. Worster, P. J. Hore, Proposal to use superparamagnetic nanoparticles to test the role of cryptochrome in magnetoreception. J. R. Soc. Interface **15**, 20180587 (2018). 30381345 10.1098/rsif.2018.0587 PMC6228473

[39] K. Kavokin, Can a hybrid chemical-ferromagnetic model of the avian compass explain its outstanding sensitivity to magnetic noise? PLoS ONE **12**, e0173887 (2017). 28296939 10.1371/journal.pone.0173887 PMC5352016

[40] S. Qin , A magnetic protein biocompass. Nat. Mater. **15**, 217–226 (2016). 26569474 10.1038/nmat4484

[41] C. Niessner, S. Denzau, L. Peichl, W. Wiltschko, R. Wiltschko, Magnetoreception: Activation of avian cryptochrome 1a in various light conditions. J. Comp. Physiol. A **204**, 977–984 (2018). 10.1007/s00359-018-1296-7 30350127

[42] R. Wiltschko, M. Ahmad, C. Niessner, D. Gehring, W. Wiltschko, Light-dependent magnetoreception in birds: The crucial step occurs in the dark. J. R. Soc. Interface **13**, 20151010 (2016). 27146685 10.1098/rsif.2015.1010 PMC4892254

[43] T. C. Player, P. J. Hore, Viability of superoxide-containing radical pairs as magnetoreceptors. J. Chem. Phys. **151**, 225101 (2019). 31837685 10.1063/1.5129608

[44] S. Y. Wong, P. Benjamin, P. J. Hore, Magnetic field effects on radical pair reactions: Estimation of B_1/2_ for flavin-tryptophan radical pairs in cryptochromes. Phys. Chem. Chem. Phys. **25**, 975–982 (2023). 36519379 10.1039/d2cp03793a PMC9811481

[45] Y. Ren, H. Hiscock, P. J. Hore, Angular precision of radical pair compass magnetoreceptors. Biophys. J. **120**, 547–555 (2021). 33421412 10.1016/j.bpj.2020.12.023 PMC7896030

[46] R. Chetverikova, G. Dautaj, L. Schwigon, K. Dedek, H. Mouritsen, Double cones in the avian retina form an oriented mosaic which might facilitate magnetoreception and/or polarized light sensing. J. R. Soc. Interface **19**, 20210877 (2022). 35414212 10.1098/rsif.2021.0877 PMC9006000

[47] S. Worster, H. Mouritsen, P. J. Hore, A light-dependent magnetoreception mechanism insensitive to light intensity and polarization. J. Roy. Soc. Interface **14**, 20170405 (2017). 28878033 10.1098/rsif.2017.0405 PMC5636276

[48] J. L. Kirschvink, M. M. Walker, “Particle size considerations for magnetite based magnetoreceptors” in Magnetite biomineralization and magnetoreception in organisms: A new biomagnetism, J. L. Kirschvink, D. S. Jones, B. J. McFadden, Eds. (Plenum Press, New York, 1985), pp. 243–254.

[49] G. Kletetschka, R. Bazala, Magnetite particle size and spatial distribution may modulate neural oscillation in the human brain. Sci. Rep. **15**, 21909 (2025). 40593272 10.1038/s41598-025-07988-2 PMC12219572

[50] D. Faivre, D. Schuler, Magnetotactic bacteria and magnetosomes. Chem. Rev. **108**, 4875–4898 (2008). 18855486 10.1021/cr078258w

[51] S. H. K. Eder , Magnetic characterization of isolated candidate vertebrate magnetoreceptor cells. Proc. Natl. Acad. Sci. U.S.A. **109**, 12022–12027 (2012). 22778440 10.1073/pnas.1205653109 PMC3409731

[52] N. B. Edelman , No evidence for intracellular magnetite in putative vertebrate magnetoreceptors identified by magnetic screening. Proc. Natl. Acad. Sci. U.S.A. **112**, 262–267 (2015). 25535350 10.1073/pnas.1407915112 PMC4291630

[53] K. Erglis , Dynamics of magnetotactic bacteria in a rotating magnetic field. Biophys. J. **93**, 1402–1412 (2007). 17526564 10.1529/biophysj.107.107474 PMC1929029

[54] B. Leberecht , Broadband 75–85 MHz radiofrequency fields disrupt magnetic compass orientation in night-migratory songbirds consistent with a flavin-based radical pair magnetoreceptor. J. Comp. Physiol. A **208**, 97–106 (2022). 10.1007/s00359-021-01537-8 PMC8918455 35019998

[55] B. Leberecht , Upper bound for broadband radiofrequency field disruption of magnetic compass orientation in night-migratory songbirds. Proc. Natl. Acad. Sci. U.S.A. **120**, 2301153120 (2023). 10.1073/pnas.2301153120 PMC10334787 37399422

[56] H. G. Hiscock, H. Mouritsen, D. E. Manolopoulos, P. J. Hore, Disruption of magnetic compass orientation in migratory birds by radiofrequency electromagnetic fields. Biophys. J. **113**, 1475–1484 (2017). 28978441 10.1016/j.bpj.2017.07.031 PMC5627152

[57] A. J. Kalmijn, R. P. Blakemore, “The magnetic behavior of mud bacteria” in Animal Migration, Navigation and Homing, K. Schmidt-Koenig, W. T. Keeton, Ed. (Springer, Berlin, Germany, 1978), pp. 354–355.

[58] W. Wiltschko, U. Munro, R. C. Beason, H. Ford, R. Wiltschko, A magnetic pulse leads to a temporary deflection in the orientation of migratory birds. Experientia **50**, 697–700 (1994).

[59] R. C. Beason, N. Dussourd, M. E. Deutschlander, Behavioral evidence for the use of magnetic material in magnetoreception by a migratory bird. J. Exp. Biol. **198**, 141–146 (1995). 9317510 10.1242/jeb.198.1.141

[60] R. C. Beason, R. Wiltschko, W. Wiltschko, Pigeon homing: Effects of magnetic pulses on initial orientation. Auk **114**, 405–415 (1997).

[61] R. A. Holland, J. L. Kirschvink, T. G. Doak, M. Wikelski, Bats use magnetite to detect the Earth’s magnetic field. PLoS ONE **3**, e1676 (2008). 18301753 10.1371/journal.pone.0001676 PMC2246016

[62] R. A. Holland, Differential effects of magnetic pulses on the orientation of naturally migrating birds. J. Roy. Soc. Interface **7**, 1617–1625 (2010). 20453067 10.1098/rsif.2010.0159 PMC2988258

[63] R. A. Holland, B. Helm, A strong magnetic pulse affects the precision of departure direction of naturally migrating adult but not juvenile birds. J. R. Soc. Interface **10**, 20121047 (2013). 23389901 10.1098/rsif.2012.1047 PMC3627120

[64] R. A. Holland, True navigation in birds: From quantum physics to global migration. J. Zool. **293**, 1–15 (2014).

[65] P. Semm, C. Demaine, Neurophysiological properties of magnetic cells in the pigeons visual-system. J. Comp. Physiol. A **159**, 619–625 (1986). 3806432 10.1007/BF00612035

[66] L. Q. Wu, J. D. Dickman, Neural correlates of a magnetic sense. Science **336**, 1054–1057 (2012). 22539554 10.1126/science.1216567

[67] M. T. Ahlers, C. T. Block, M. Winklhofer, M. Greschner, Integration and evaluation of magnetic stimulation in physiology setups. PLoS ONE **17**, e0271765 (2022). 35867646 10.1371/journal.pone.0271765 PMC9307166

[68] S. Nimpf, H. S. Kaplan, G. C. Nordmann, T. Cushion, D. A. Keays, Long-term, high-resolution in vivo calcium imaging in pigeons. Cell Rep. Methods **4**, 100711 (2024). 38382523 10.1016/j.crmeth.2024.100711 PMC10921020

[69] T. Baden , The functional diversity of retinal ganglion cells in the mouse. Nature **529**, 345–350 (2016). 26735013 10.1038/nature16468 PMC4724341

[70] K. Franke , Inhibition decorrelates visual feature representations in the inner retina. Nature **542**, 439–444 (2017). 28178238 10.1038/nature21394 PMC5325673

[71] S. Weiler , High-yield in vitro recordings from neurons functionally characterized in vivo. Nat. Protoc. **13**, 1275–1293 (2018). 29748648 10.1038/nprot.2018.026

[72] D. R. Kattnig , Chemical amplification of magnetic field effects relevant to avian magnetoreception. Nat. Chem. **8**, 384–391 (2016). 27001735 10.1038/nchem.2447

[73] C. A. Dodson , Fluorescence-detected magnetic field effects on radical pair reactions from femtolitre volumes. Chem. Commun. **51**, 8023–8026 (2015). 10.1039/c5cc01099c 25865161

[74] C. R. Timmel, U. Till, B. Brocklehurst, K. A. McLauchlan, P. J. Hore, Effects of weak magnetic fields on free radical recombination reactions. Mol. Phys. **95**, 71–89 (1998). 10.1080/09553000050176270 11098854

[75] G. J. Pazera, T. P. Fay, I. A. Solov’yov, P. J. Hore, L. Gerhards, Spin dynamics of radical pairs using the stochastic Schrödinger equation in MolSpin. J. Chem. Theory Comput. **20**, 8412–8421 (2024). 39283312 10.1021/acs.jctc.4c00361 PMC11465467

[76] T. P. Fay, L. P. Lindoy, D. E. Manolopoulos, Spin relaxation in radical pairs from the stochastic Schrodinger equation. J. Chem. Phys. **154**, 084121 (2021). 33639770 10.1063/5.0040519

