# Supplementary material for: A hybrid compass mechanism combining radical pairs and magnetite crystals
Source: Proc Natl Acad Sci U S A. 2026 Feb 19;123(8):e2524093123. doi: 10.1073/pnas.2524093123 (PMC12933104; doi:10.1073/pnas.2524093123)
Supplement: Supplementary file 1 — Appendix 01 (PDF) [file pnas.2524093123.sapp.pdf]

A hybrid compass mechanism combining radical pairs and magnetite crystals

P. J. Hore

Department of Chemistry, University of Oxford, Oxford, OX1 3QZ, UK

Supplementary Information

Contents

S1. Spin dynamics simulations: toy FAD-TrpH radical pairs.......... 1
S2. Spin dynamics simulations: more realistic FAD-TrpH radical pairs.......... 2
S3. Hybrid model simulations.......... 3
S4. Differential effects of spin relaxation.......... 4
S5. Number of radical pairs required for a given angular precision.......... 5
S6. Sensitivity differences between standard and hybrid models.......... 6
S7. Dipolar coupling.......... 7
S8. Magnetic field gradients.......... 7
S9. Finite pool of CRY molecules.......... 8
S10. Time taken for a magnetic nanoparticle to align in a 50-μT magnetic field.......... 8
S11. Radiofrequency field effects.......... 9
S12. Comments on possible behavioural tests to distinguish the two models.......... 10

References.......... 11

S1. Spin dynamics simulations: toy FAD-TrpH radical pairs

The data in Figure 1 were calculated as described in (1). The initial state of the radical pair was pure singlet. Singlet and triplet radical pairs reacted to give distinct products with first order rate constants $k_S = k_T = 10^6\ s^{-1}$. Spin relaxation was not included. The g-values of both radicals were 2.0023. The fractional triplet yield was calculated for 400 magnetic-field directions, uniformly distributed over a hemisphere. The invariance of Φ_T to exact inversion of the magnetic field ($\mathbf{B}_{GMF} \rightarrow -\mathbf{B}_{GMF}$) means that full spherical averaging is redundant (2, 3). Hyperfine and dipolar coupling tensors and atom labels can be found in Sections S2 and S3 of the Supplementary Information of (4). The hyperfine interactions included in each of the panels of Figure 1 are given in Table S1 (F = $FAD^{\bullet-}$, W = $TrpH^{\bullet+}$).

Table S1. Hyperfine interactions used for Figure 1.

(a)	(b)	(c)	(d)
FN5	FN5	FN5	FN5
FN10	FN10	FN10	FN10
	WNE1	WNE1	WNE1
		WHB1	WHB1
			WHE1

S2. Spin dynamics simulations: more realistic FAD-TrpH radical pairs

Calculations were performed for two larger spin systems (with $n_{\text{nuc}} = 8$ and $n_{\text{nuc}} = 14$ nuclear spins) using *MolSpin* (5, 6). The hyperfine interactions (taken from the Supplementary Information of (4)) were:

$n_{\text{nuc}} = 8$: FN5, FN10, FH6, FH1′, WNE1, WHE1, WHE3, WHZ2.

$n_{\text{nuc}} = 14$: FN5, FN10, FH6, FH1′, FH8, FH7, FH9, WNE1, WHE1, WHE3, WHZ2, WHH2, WHD1, WHB1.

The simulation methods used were:

$n_{\text{nuc}} = 8$: Exact solution of the Liouville von Neumann equation using the “Direct method”, as described in (6).

$n_{\text{nuc}} = 14$: Stochastic Schrödinger equation method with M = 6 SU(Z) states, as described in (5-7).

In both cases, the system was propagated with 1-ns time steps from t = 0 to t = 7 μs. Other conditions: as for Figure 1 (*Section S1*). Triplet yields were calculated for magnetic fields of strength 50 μT and 50 mT, with the field parallel to either the X-axis or the Z-axis of the $\text{FAD}^{\bullet-}$ radical. The flavin axis system is shown in Figure 1 of (6). The results are given in Table S2.

Table S2. Magnetic field effects for FAD-TrpH radical pairs.

	$n_{\text{nuc}} = 8$		$n_{\text{nuc}} = 14$	
B	50 μT	50 mT	50 μT	50 mT
$\Phi_{\text{T}}(X,B)$	0.709299	0.489134	0.729479	0.490177
$\Phi_{\text{T}}(Z,B)$	0.709075	0.466559	0.729394	0.466790
$\Delta\Phi_{\text{T}}(B)$	0.000224	0.022575	0.000085	0.023327
$\Phi_{\text{T}}(0), \Phi_{\text{T}}(\infty)$	0.709187	0.477847	0.729437	0.478454

Standard model: $\Delta\Phi_{\text{T}}(B) = \Phi_{\text{T}}(X,B) - \Phi_{\text{T}}(Z,B)$

In Equation (2): $\Phi_{\text{T}}(0) \approx \frac{1}{2}\Phi_{\text{T}}(X, 50\,\mu\text{T}) + \frac{1}{2}\Phi_{\text{T}}(Z, 50\,\mu\text{T})$

In Equation (2): $\Phi_{\text{T}}(\infty) \approx \frac{1}{2}\Phi_{\text{T}}(X, 50\,\text{mT}) + \frac{1}{2}\Phi_{\text{T}}(Z, 50\,\text{mT})$

The data in Table S2 were kindly provided by Dr Luca Gerhards, University of Oldenburg.

S3. Hybrid model simulations

The information in Figures 3 and 4 was calculated as follows. The position of the radical pairs relative to the centre of the nanoparticle, the geomagnetic field and the magnetic dipole moment of the nanoparticle were defined as

$$\mathbf{r} = r\left(\hat{\mathbf{x}}\sin\theta + \hat{\mathbf{z}}\cos\theta\right), \tag{S1}$$

$$\mathbf{B}_{\mathrm{GMF}} = B_{\mathrm{GMF}}\hat{\mathbf{z}}, \tag{S2}$$

$$\boldsymbol{\mu}_{\mathrm{MNP}} = \mu_{\mathrm{MNP}}\left(\hat{\mathbf{x}}\sin\zeta\cos\nu + \hat{\mathbf{y}}\sin\zeta\sin\nu + \hat{\mathbf{z}}\cos\zeta\right), \tag{S3}$$

where $\hat{\mathbf{x}}$, $\hat{\mathbf{y}}$, and $\hat{\mathbf{z}}$ are orthogonal unit vectors, B_{GMF} = 50 μT, and

$$\mu_{\mathrm{MNP}} = \frac{4\pi R^3}{3} M_{\mathrm{S}}, \tag{S4}$$

with $M_{\mathrm{S}} = 4.8 \times 10^5$ A m^{-1}, the saturation magnetization of magnetite (8). The magnetic energy of the nanoparticle in the geomagnetic field is

$$U = -\boldsymbol{\mu}_{\mathrm{MNP}} \cdot \mathbf{B}_{\mathrm{GMF}} = -\mu_{\mathrm{MNP}} B_{\mathrm{GMF}} \cos\zeta, \tag{S5}$$

and the probability distribution of magnetic moment directions is

$$p(\zeta) = \frac{\mathrm{e}^{-U/k_{\mathrm{B}}T}}{\int_0^{2\pi}\mathrm{d}\nu \int_0^{\pi}\mathrm{d}\zeta\, \mathrm{e}^{-U/k_{\mathrm{B}}T}\sin\zeta} = \frac{\eta\,\mathrm{e}^{\eta\cos\zeta}}{4\pi\sinh(\eta)} \tag{S6}$$

with

$$\eta = \mu_{\mathrm{MNP}} B_{\mathrm{GMF}} / k_{\mathrm{B}}T. \tag{S7}$$

The magnetic field generated by the nanoparticle at position **r** is

$$\mathbf{B}_{\mathrm{MNP}}(\mathbf{r}) = \left(\frac{\mu_0}{4\pi}\right)\left(-\frac{\boldsymbol{\mu}_{\mathrm{MNP}}}{r^3} + \frac{3(\boldsymbol{\mu}_{\mathrm{MNP}} \cdot \mathbf{r})\mathbf{r}}{r^5}\right) \tag{S8}$$

and the total field at **r** is

$$B_{\mathrm{total}}(r,\theta) = \left|\mathbf{B}_{\mathrm{GMF}} + \mathbf{B}_{\mathrm{MNP}}(\mathbf{r})\right|. \tag{S9}$$

The anisotropy of the triplet yield is

$$\Delta\Phi_{\mathrm{T}} = \left\langle \Phi_{\mathrm{T}}\left(B_{\mathrm{total}}(r,90°)\right)\right\rangle - \left\langle \Phi_{\mathrm{T}}\left(B_{\mathrm{total}}(r,0)\right)\right\rangle \tag{S10}$$

where

$$\langle \cdots \rangle = \int_0^{2\pi}\mathrm{d}\nu \int_0^{\pi}\mathrm{d}\zeta\,(\cdots)\,p(\zeta)\sin\zeta \tag{S11}$$

and

$$\Phi_{\mathrm{T}}(B) = \Phi_{\mathrm{T}}(\infty) + \frac{\Phi_{\mathrm{T}}(0) - \Phi_{\mathrm{T}}(\infty)}{1 + \left(B/B_{1/2}\right)^2}. \tag{S12}$$

The probability distributions, $p(\zeta)$, Equation [S6], for the orientation of spherical magnetite nanoparticles with radii R = 30-70 nm in a B_{GMF} = 50 μT magnetic field are shown in Figure S1. The corresponding values of η at 40 °C are 0.63, 1.49, 2.91, 5.02, and 7.98.

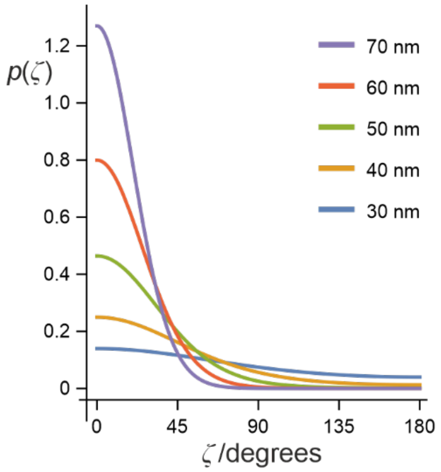

Figure S1. Probability distributions of the magnetic moment directions, $p(\zeta)$.

The dependence of $\Delta\Phi_{\mathrm{T}}^{\max}$ on μ_{MNP} in Figure 3f is given by the approximate empirical expression:

$$\ln\left(\Delta\Phi_{\mathrm{T}}^{\max}\right) \approx -2.48+\frac{0.951(\ln x-2.17)}{1+9.17x^{1.04}}, \qquad \text{[S13]}$$

where

$$x=\mu_{\mathrm{MNP}} / \mathrm{fA\ m}^2 . \qquad \text{[S14]}$$

S4. Differential effects of spin relaxation

The feeble dependence of the reaction yield on the direction of a 50 μT magnetic field is likely to be exacerbated by spin relaxation (which was not included in Figure 1). Figure S2 shows simulations of the 5-nucleus toy model (used for Figure 1d) in which the electron spins relax towards equilibrium with a rate constant w (in the range 0 to 10^8 s^{-1}) while reacting (recombining) from both singlet and triplet states with the same rate constant k (in the range 10^5 to 10^8 s^{-1}). Apart from the inclusion of spin relaxation and the variable rate constant, the conditions are identical to those used for Figure 1d. The calculations were carried out as described in (9).

The quantities plotted here are (a) the isotropic effect of a 50 mT field, i.e. $\Phi_{\mathrm{T}}(50\ \mathrm{mT})-\Phi_{\mathrm{T}}(0)$, averaged over the 400 magnetic field directions, and (b) the anisotropic effect of a 50 μT field (i.e. maximum-minus-minimum value of $\Phi_{\mathrm{T}}(50\ \mu\mathrm{T})$). In both cases, as expected, the maximum signal is found when the radicals recombine and relax slowly (k = 10^5 s^{-1}, w = 0). In line with Figure 1d, the high-field isotropic signal in (a) is much larger than the low-field anisotropic signal in (b). The other difference between the two calculations is that while both signals are attenuated by faster reaction and faster relaxation, the 50 mT signal is much less affected by the former than by the latter. For example,

when $k = w = 10^7$ s^{-1}, the signal in (a) is 45% of that for slow recombination and no relaxation ($k = 10^5$ s^{-1}, $w = 0$). For (b), the corresponding number is 5%. If, *in vivo*, spin relaxation occurs with a rate constant of 10^7 s^{-1} then the high-field isotropic signal can be rescued by increasing the recombination rate constant from 10^5 s^{-1} to 10^7 s^{-1} or 10^8 s^{-1}, an option that does not exist for the low-field anisotropic signal.

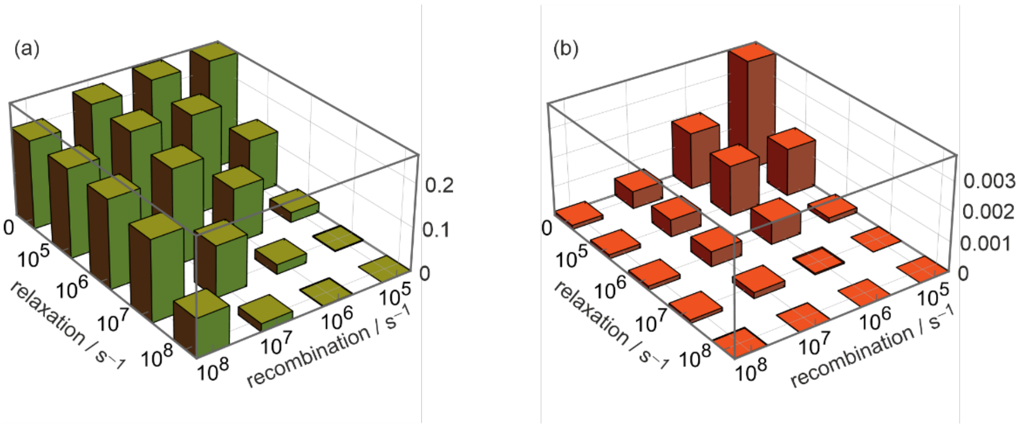

Figure S2. (a) The isotropic effect of a 50 mT field, i.e. $\Phi_T(50\text{ mT}) - \Phi_T(0)$. (b) The anisotropic effect of a 50 μT field (i.e. maximum-minus-minimum value of $\Phi_T(50\ \mu\text{T})$).

S5. Number of radical pairs required for a given angular precision

Information theory can be used to obtain a strict lower bound on the angular precision with which a bird could orient itself using only geomagnetic cues derived from a radical-pair sensor (10, 11). With this approach, one can estimate the minimum number of radical pairs (n_{RP}) needed to detect the direction of the geomagnetic field to within 5° for a given value of $\Delta\Phi_T$ (in Equation [4] in the main text). Modelling the dependence of the triplet yield on the magnetic field direction as

$$\Phi_T(\gamma) = 0.5 - \Delta\Phi_T(\cos^2\gamma - \tfrac{1}{3}) \qquad [S15]$$

we find that n_{RP} is inversely proportional to $\Delta\Phi_T^2$ as shown in Figure S3 for three orientations (O1-O3) of the cryptochromes within the photoreceptor cells (defined in (11)).

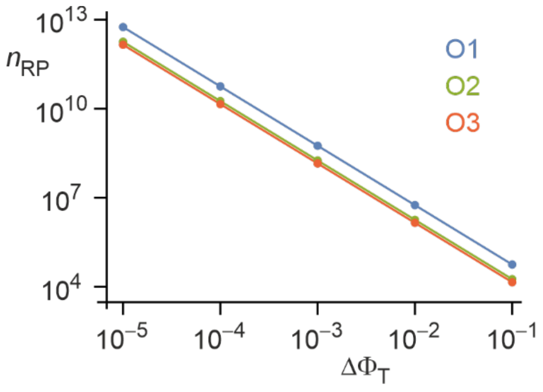

Figure S3. Dependence of n_{RP} on $\Delta\Phi_T$.

Table S3 gives calculated values of n_{RP} for these three orientations when $\Delta\Phi_T = 8.5 \times 10^{-5}$ (standard model) and $\Delta\Phi_T = 3.0 \times 10^{-2}$ (hybrid model).

Table S3. Estimates of the number of radical pairs (n_{RP}) needed to detect the direction of the geomagnetic field to within 5°.

	standard	hybrid
$\Delta\Phi_T$	8.5×10^{-5}	3.0×10^{-2}
O1	7.77×10^{10}	6.13×10^{5}
O2	2.48×10^{10}	1.99×10^{5}
O3	1.97×10^{10}	1.58×10^{5}

Based on these figures, a hybrid compass could have the same precision as the standard model with ~10^5 fewer radical pairs and therefore photons. The number of photons that enter the double-cone photoreceptor cells in a songbird's eye on a clear, moonless, star-lit night is ~2×10^8 min^{-1} (10) (an estimate based on ~3×10^6 double cones in the retina and ~1 photon s^{-1} per double cone). Assuming each photon that enters a double cone produces a radical pair, this number is roughly 3 orders of magnitude more than would be required for the hybrid compass and about 2 orders of magnitude fewer than needed by a standard-model compass.

A completely different method (12), based on the "energy resolution limit", has been used to estimate that the minimum number of CRY4a molecules a bird would need for a functional radical pair compass is between 2×10^4 and 2×10^6. Given the large number of assumptions and approximations involved in both estimates, the apparent agreement with the final column of Table S3 is very probably fortuitous.

S6. Sensitivity differences between standard and hybrid models

The differences in the sensitivity of the two models have their origins in the constraints on the properties of the radical pairs and the proteins that contain them (13). In the standard model, to be optimally sensitive to the direction of the geomagnetic field, several conditions should be satisfied: (a) there should be a small number of strongly anisotropic hyperfine interactions, ideally with mutually aligned principal axes; (b) the electron-electron dipolar interaction should be small, ideally much less than 50 μT; (c) the radical pairs should live for at least 1 μs; (d) spin relaxation should be much slower than radical-pair recombination; and (e) the protein molecules should be perfectly mutually aligned and immobilized. The $FAD^{\bullet-}$ $TrpH^{\bullet+}$ radical pair in CRY4a clearly does not satisfy conditions (a) or (b) which is why the triplet anisotropy calculated above is as small as 8.5×10^{-5}. Furthermore, conditions (d) and (e) have been assumed in the above calculations but are probably unrealistic *in vivo*. While some of these constraints may not apply in some of the proposed variants of the standard model (14-16), or could be alleviated by evolutionary optimisation, others would be more difficult to circumvent. The nature of these five conditions probably accounts for the failure, so far, to detect effects of < 1 mT

magnetic fields on the photochemistry of purified cryptochromes, despite the development of bespoke, state-of-the-art spectroscopic methods and careful optimisation of sample conditions (17, 18).

The hybrid mechanism, although still reliant on radical pairs for magnetic sensing, relaxes many of the constraints of the standard model. Instead of directly detecting the tiny (~100 ppm) dependence of the yield of the radical pair reaction on the direction of the Earth's magnetic field, it uses a magnetic particle to amplify the Earth's field in such a way that the radical pairs are required to respond to the *intensity*, rather than the direction, of a *much stronger* magnetic field. The isotropic effects of magnetic fields comparable to the hyperfine interactions are always much larger than the anisotropic effects of magnetic fields much weaker than most of the hyperfine interactions (Figure 1). Referring to the five conditions in the last paragraph: (a) partial cancellation of the effects of anisotropic hyperfine interactions is no longer an issue when the crucial quantity is the rotationally averaged magnetic field effect; (b) the dipolar interaction does much less harm; (c) the radical pairs do not have to persist for as long as 1 μs; (d) spin relaxation can be less of a problem; and (e) it is no longer essential that the proteins are rigidly immobilized and rotationally ordered. Points (b)-(d) are elaborated in *Sections S4 and S7.*

S7. Dipolar coupling

Figure S4 shows the effects of dipolar coupling on the triplet yield of the $(n_{\text{FAD}}, n_{\text{TrpH}}) = (2,3)$ toy radical pair in Figure 1d. (a) is identical to Figure 1d and was calculated with a dipolar tensor appropriate for FAD and Trp318 in pigeon CRY4a (19) (dipolar coupling parameter, D = –0.511 mT). The calculation in (b) is identical to (a) except that the dipolar interaction has been omitted.

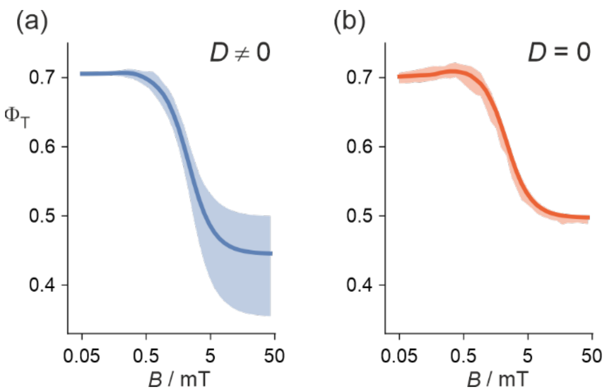

Figure S4. Magnetic field effects (a) with and (b) without dipolar coupling.

The average isotropic magnetic field effect is not strongly affected by the dipolar coupling: $\Delta\Phi_{\text{T}}(0) - \Delta\Phi_{\text{T}}(\infty)$ = 0.260 with dipolar coupling (a) and 0.186 without (b).

S8. Magnetic field gradients

Previous discussions of the effects of nanomagnets on radical pair reactions have concentrated on singlet-triplet interconversion driven by the gradient of the fringing field (20, 21). If the particle is small and the radical pairs are close to its surface, the difference in the magnetic fields experienced by the

two radicals, ΔB , can dominate the hyperfine interactions and boost the magnetic sensitivity. Magnetic field gradients are not expected to be important here.

The fringing field (in tesla) of a spherical magnetite particle of radius R , a distance r from its centre is

$$B_{\mathrm{MNP}} = \frac{2\mu_{\mathrm{MNP}}}{r^3}\frac{\mu_0}{4\pi} = \frac{2\mu_0 M_{\mathrm{S}}}{3}\frac{R^3}{r^3} = 0.402\frac{R^3}{r^3} \quad \text{[S16]}$$

when $\theta = 0$ and half that value when $\theta = 90°$ (θ being the angle between $\boldsymbol{\mu}_{\mathrm{MNP}}$ and $\mathbf{r}$). The magnetic field gradient at a distance $r = 4R$ (where $\Delta\Phi_{\mathrm{T}}$ is close to a maximum, Figure 3d) is

$$\frac{\mathrm{d}B_{\mathrm{MNP}}}{\mathrm{d}r} = -\frac{4.71\times10^{-3}}{R} \quad \text{[S17]}$$

with r and R in metres, and B_{MNP} in tesla.

The maximum difference in the magnetic fields experienced by two radicals 2 nm apart (the separation of $FAD^{\bullet-}$ and $TrpH^{\bullet+}$ in CRY4a (19)) is therefore 0.31 mT when R = 30 nm and 0.13 mT when R = 70 nm. These values are small compared to the effective hyperfine interaction in $FAD^{\bullet-}$ $TrpH^{\bullet+}$ ($B_{1/2}$ ~ 4 mT) and so should have a minor influence on the spin dynamics.

S9. Finite pool of CRY molecules

In the case that the radical pairs occupy a spherical region of radius ρ , Equation [S11] was replaced by:

$$\langle\cdots\rangle = \frac{1}{V_\rho}\int_{sphere}\int_0^{2\pi}\mathrm{d}v\int_0^{\pi}\mathrm{d}\zeta\,(\cdots)\,p(\zeta)\sin\zeta \quad \text{[S18]}$$

where *sphere* is a spherical region of radius ρ centred at $\mathbf{r} = r\hat{\mathbf{z}}$ when $\theta = 0$ and at $\mathbf{r} = r\hat{\mathbf{x}}$ when $\theta = 90°$. $V_\rho = 4\pi\rho^3/3$. The results for R = 50 nm and r = 210 nm are given in Table S4.

Table S4. Magnetic field effects for radical pairs occupying a spherical region of radius ρ .

ρ / nm	$\Delta\Phi_{\mathrm{T}}(\rho)$	$\Delta\Phi_{\mathrm{T}}(\rho)/\Delta\Phi_{\mathrm{T}}(0)$
0	0.0596	1
25	0.0578	0.97
50	0.0529	0.89
100	0.0392	0.66

S10. Time taken for a magnetic nanoparticle to align in a 50-µT magnetic field

Following Erglis et al. (22), the time required for a spherical nanoparticle (magnetic dipole moment $\boldsymbol{\mu}_{\mathrm{MNP}}$, radius R, saturation magnetization M_{S}) to rotate into alignment with a magnetic field ($\mathbf{B}$) can be estimated by considering the balance between magnetic and viscous torques:

$$\boldsymbol{\mu}_{\mathrm{MNP}} \times \mathbf{B} = C\eta\boldsymbol{\omega}, \quad \text{[S19]}$$

where $C = 8\pi R^3$ is the friction coefficient, η is the viscosity of the surrounding medium, and $\boldsymbol{\omega}$ is the angular velocity of the particle. Defining $\hat{\mathbf{x}}, \hat{\mathbf{y}}, \hat{\mathbf{z}}$ as orthogonal unit vectors and

$$\boldsymbol{\mu}_{\text{MNP}} = \mu_{\text{MNP}}\left(\cos\theta\,\hat{\mathbf{x}} + \sin\theta\,\hat{\mathbf{y}}\right) = \frac{4\pi R^3 M_{\text{S}}}{3}\left(\cos\theta\,\hat{\mathbf{x}} + \sin\theta\,\hat{\mathbf{y}}\right) \qquad \text{[S20]}$$

$$\mathbf{B} = B\hat{\mathbf{x}} \qquad \text{[S21]}$$

$$\boldsymbol{\omega} = \frac{\mathrm{d}\theta}{\mathrm{d}t}\hat{\mathbf{z}} \qquad \text{[S22]}$$

$$\mu = 4\pi R^3 M_{\text{S}} / 3 \qquad \text{[S23]}$$

we obtain

$$\frac{\mathrm{d}\theta}{\mathrm{d}t} = -\frac{1}{\tau}\sin\theta \quad \text{with} \quad \tau = \frac{6\eta}{M_{\text{S}}B} \qquad \text{[S24]}$$

and hence, taking $\theta(0) = \pi/2$:

$$\theta(t) = \cos^{-1}[\tanh(t/\tau)]\,. \qquad \text{[S25]}$$

Using $\eta = 6.5 \times 10^{-4}$ J s m^{-3} (water at 40 °C), $B = 50$ µT, and $M_{\text{S}} = 4.8 \times 10^5$ A m^{-1} gives $\tau = 163$ µs. Figure S5 shows $\theta(t)$ for media with the viscosity 1, 5, and 20 times that of water at 40 °C.

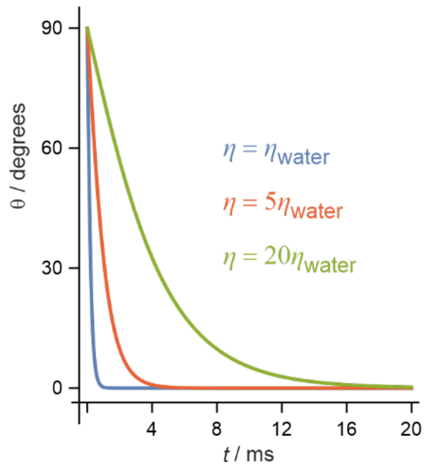

Figure S5. Time dependence of the alignment of a magnetic nanoparticle in the geomagnetic field.

S11. Radiofrequency field effects

The likely effects of time-dependent magnetic fields on a radical pair can be gleaned from its "action-spectrum histogram" (23). Calculated from the hyperfine, dipolar and Zeeman interactions, these graphs show the relative change in the reaction yield of radical pairs subject to weak radiofrequency fields in resonance with spin transitions in the radicals: the taller the histogram bar, the larger the expected effect at that frequency.

In a 50-µT static magnetic field, the action-spectrum histogram is dominated by hyperfine and dipolar interactions and shows a threshold or "cut-off" frequency beyond which there are no radiofrequency field effects (23). For the $FAD^{\bullet-}$ $TrpH^{\bullet+}$ radical pair, with 27 hyperfine interactions, this

cut-off comes at 116 MHz (24, 25), corresponding to the gap between the highest- and lowest-energy eigenstates of the spin Hamiltonian.

Figure S6 shows action-spectrum histograms for the toy radical pair (Figure 1d) with $(n_{\mathrm{FAD}}, n_{\mathrm{TrpH}})$ = (2, 3). In the geomagnetic field, panel (a), there are resonances up to the ~80 MHz cut-off frequency for this smaller spin system with just 5 hyperfine interactions. As the static field is increased through the range relevant for the hybrid model (2-8 mT, (b)-(e)), the Zeeman interaction dominates and the band of resonance frequencies becomes centred at the electron Larmor frequency, $\nu_{\mathrm{L}} = \gamma_{\mathrm{e}} B / 2\pi$. When B = 8 mT, for example, ν_{L} = 224 MHz. Under these conditions, the allowed transitions are between the $M_S = 0$ and $M_S = \pm 1$ spin states of the radical pair.

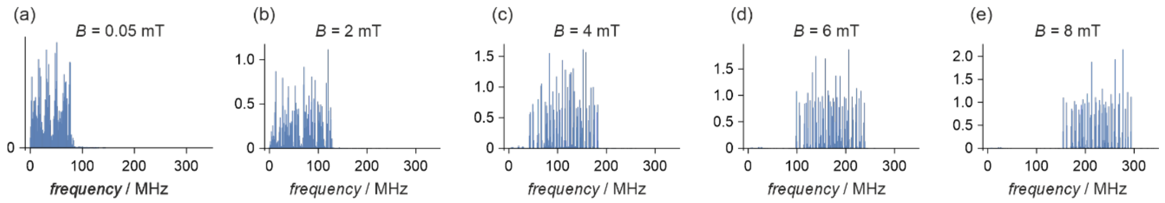

Figure S6. Action spectrum histograms for static magnetic fields: (a) 0.05 mT; (b) 2.0 mT, (c) 4.0 mT; (d) 6.0 mT; (e) 8.0 mT.

S12. Comments on possible behavioural tests to distinguish the two models

Both standard and hybrid models have cryptochrome as the sensor, making it challenging to devise a behavioural test to distinguish them. RNA interference techniques (26) aimed at reducing the expression of CRY4a in the avian retina would disrupt both types of magnetoreceptor. Accelerating the loss of spin coherence in CRY4a molecules *in vivo* by attaching superparamagnetic nanoparticles (27) would also disable both mechanisms.

Furthermore, it would be challenging to devise a behavioural test to probe the magnetic field dependence predicted by Figure 4. It appears that birds cannot spontaneously use their magnetic compass when tested in magnetic fields about 30% weaker or stronger than the local field (28). Although pre-exposure of birds for periods of a day or more to magnetic fields up to 150 μT (29) (or down to 4 μT (30)) can expand this range, it seems unlikely that pre-exposure would be successful for the ~3 mT fields needed to detect the hybrid-sensor peak in Figure 4a.

References

1. C. R. Timmel, U. Till, B. Brocklehurst, K. A. McLauchlan, P. J. Hore, Effects of weak magnetic fields on free radical recombination reactions. *Molec. Phys.* **95**, 71–89 (1998).
2. A. Lewis, *Spin Dynamics in Radical Pairs* (Springer International Publishing, 2018).
3. P. L. Benjamin, L. Gerhards, I. A. Solov'yov, P. J. Hore, Magnetosensitivity of model flavin-tryptophan radical pairs in a dynamic protein environment. *J. Phys. Chem. B* **129**, 5937–5947 (2025).
4. S. Y. Wong, P. Benjamin, P. J. Hore, Magnetic field effects on radical pair reactions: estimation of $B_{1/2}$ for flavin-tryptophan radical pairs in cryptochromes. *Phys. Chem. Chem. Phys.* **25**, 975–982 (2023).
5. L. Gerhards, C. Nielsen, D. R. Kattnig, P. J. Hore, I. A. Solov'yov, Modeling spin relaxation in complex radical systems using *MolSpin*. *J. Comput. Chem.* **44**, 1704–1714 (2023).
6. G. J. Pazera, T. P. Fay, I. A. Solov'yov, P. J. Hore, L. Gerhards, Spin dynamics of radical pairs using the stochastic Schrödinger equation in *MolSpin*. *J. Chem. Theory Comput.* **20**, 8412–8421 (2024).
7. T. P. Fay, L. P. Lindoy, D. E. Manolopoulos, Spin relaxation in radical pairs from the stochastic Schrodinger equation. *J. Chem. Phys.* **154**, 084121 (2021).
8. V. Binhi, Do naturally occurring magnetic nanoparticles in the human body mediate increased risk of childhood leukaemia with EMF exposure? *Int. J. Radiat. Biol.* **84**, 569–579 (2008).
9. P. J. Hore, Upper bound on the biological effects of 50/60 Hz magnetic fields mediated by radical pairs. *eLife* **8**, e44179 (2019).
10. H. G. Hiscock *et al.*, Navigating at night: fundamental limits on the sensitivity of radical pair magnetoreception under dim light. *Q. Rev. Biophys.* **52**, e9 (2019).
11. Y. Ren, H. Hiscock, P. J. Hore, Angular precision of radical pair compass magnetoreceptors. *Biophys. J.* **120**, 547–555 (2021).
12. I. K. Kominis, E. Gkoudinakis, Approaching the quantum limit of energy resolution in animal nagnetoreception. *PRX LIFE* **3**, 013004 (2025).
13. P. J. Hore, H. Mouritsen, The radical pair mechanism of magnetoreception. *Annu. Rev. Biophys.* **45**, 299–344 (2016).
14. N. S. Babcock, D. R. Kattnig, Radical scavenging could answer the challenge posed by electron-electron dipolar interactions in the cryptochrome compass model. *JACS Au* **1**, 2033–2046 (2021).
15. M. C. J. Denton *et al.*, Magnetosensitivity of tightly bound radical pairs in cryptochrome is enabled by the quantum Zeno effect. *Nat. Comm.* **15**, 10823 (2024).
16. D. R. Kattnig, Radical-pair-based magnetoreception amplified by radical scavenging: resilience to spin relaxation. *J. Phys. Chem. B* **121**, 10215–10227 (2017).
17. J. Xu *et al.*, Magnetic sensitivity of cryptochrome 4 from a migratory songbird. *Nature* **594**, 535–540 (2021).
18. J. Gravell *et al.*, Spectroscopic characterisation of radical pair photochemistry in nonmigratory avian cryptochromes: magnetic field effects in *Gg*Cry4a. *J. Am. Chem. Soc.* **147**, 24286–24298 (2025).
19. B. D. Zoltowski *et al.*, Chemical and structural analysis of a photoactive vertebrate cryptochrome from pigeon. *Proc. Natl. Acad. Sci. USA* **116**, 19449–19457 (2019).
20. J. Cai, Quantum probe and design for a chemical compass with magnetic nanostructures. *Phys. Rev. Lett.* **106**, 100501 (2011).
21. A. E. Cohen, Nanomagnetic control of intersystem crossing. *J. Phys. Chem. A* **113**, 11084–11092 (2009).
22. K. Erglis *et al.*, Dynamics of magnetotactic bacteria in a rotating magnetic field. *Biophys. J.* **93**, 1402–1412 (2007).
23. H. G. Hiscock, H. Mouritsen, D. E. Manolopoulos, P. J. Hore, Disruption of magnetic compass orientation in migratory birds by radiofrequency electromagnetic fields. *Biophys. J.* **113**, 1475–1484 (2017).

24. B. Leberecht *et al.*, Broadband 75-85 MHz radiofrequency fields disrupt magnetic compass orientation in night-migratory songbirds consistent with a flavin-based radical pair magnetoreceptor. *J. Comp. Physiol. A* **208**, 97–106 (2022).
25. B. Leberecht *et al.*, Upper bound for broadband radiofrequency field disruption of magnetic compass orientation in night-migratory songbirds. *Proc. Natl. Acad. Sci. USA* **120**, 2301153120 (2023).
26. P. K. Seth *et al.*, AAV-mediated transduction of songbird retina. *Front. Physiol.* **16**, 1549585. (2025).
27. S. B. Worster, P. J. Hore, Proposal to use superparamagnetic nanoparticles to test the role of cryptochrome in magnetoreception. *J. R. Soc. Interface* **15**, 20180587 (2018).
28. W. Wiltschko, Über den Einfluß statischer Magnetfelder auf die Zugorientierung der Rotkehlchen (*Erithacus rubecula*). *Z. Tierpsychol.* **25**, 537–558 (1968).
29. W. Wiltschko, "Further analysis of the magnetic compass of migratory birds" in Animal migration, navigation, and homing, K. Schmidt-Koenig, W. T. Keeton, Eds. (Springer, Berlin, Germany, 1978), pp. 302–310.
30. M. Winklhofer, E. Dylda, P. Thalau, W. Wiltschko, R. Wiltschko, Avian magnetic compass can be tuned to anomalously low magnetic intensities. *Proc. R. Soc. B* **280**, 20130853 (2013).
